# Surgical Management of Primary Bone Lymphoma of the Hip: A Case Report and Review of the Literature

**DOI:** 10.1155/2019/3174768

**Published:** 2019-03-03

**Authors:** Grace Kennedy, Phil Weir, Kevin Johnston, Patrick Elder

**Affiliations:** Department of Haematology, Altnagelvin Area Hospital, Glenshane Road, Londonderry, Co. Londonderry, Ireland

## Abstract

**Introduction:**

Primary bone lymphoma (PBL) is a rare bone malignancy which may present with atraumatic pain, swelling, or pathological fracture. Whilst the femur is the most commonly affected site, any bone may be involved. PBL should be distinguished from other bone lesions to determine clinical management.

**Case Report:**

We report the case of an 89-year-old gentleman who presented to the local emergency department with atraumatic hip pain and inability to weight-bear. Multimodal imaging showed evidence of a tumor involving the proximal femur and adjacent acetabulum with an associated pathological intertrochanteric fracture. Biopsy specimens demonstrated this to be PBL of the diffuse large B-cell subtype. No other disease foci or nodal involvement was identified. The patient underwent proximal femoral replacement and acetabular reconstruction prior to commencing R-Mini-CHOP chemotherapy, during which time he has been permitted to fully weight-bear.

**Conclusion:**

To our knowledge, this is the first reported case of a patient having PBL with both femoral and acetabular involvements. Due to its infrequent occurrence, evidence remains limited to advise therapeutic guidelines. Our practice concurs with literature suggesting that surgery be reserved for cases of pathological fracture. However, the merits of undergoing surgical fixation prior to chemoradiation treatment have been considered.

## 1. Introduction

First described by Oberling in 1928 [1], primary bone lymphoma (PBL) is a rare clinical entity accounting for approximately 5% of all localised extranodal lymphomas [2]. Most PBL are of the non-Hodgkin class, and diffuse large B-cell lymphoma (DLBCL) is by far (>85%) the most common subtype [3]. The definition of PBL varies within the literature. Generally, PBL is regarded as a malignant lymphoma arising within the medullary cavity of a single bone *without* nodal or visceral involvement. However, other authors have extended this definition to include cases of regional lymph node involvement appearing six months *after* the onset of the symptoms of the primary disease focus [4].

PBL exhibits a slight male preponderance, with a male to female ratio of 1.5 : 1 [5]. PBL typically affects those in the fifth to seventh decades of life, with median age at presentation reported in the remit of 48-55 years [6, 7]. Most patients have limited-stage disease, usually Ann Arbour stage IE or IIE, at the time of presentation [8, 9], with solitary lesion disease reported in approximately 90% of patients [7].

PBL most commonly affects the femur; however, other skeletal sites may be involved including the innominatum, tibia, fibula, humerus, spine, mandible, radius, scapula, skull, talus, and synovium associated with the knee [6, 10, 11]. Patients with PBL most commonly present with pain but may also present with a fracture, localised swelling, or suspected periprosthetic joint infection [6, 12]. Up to one-fifth of patients may present with pathological fracture, and this has been associated with poorer five-year overall and progression-free survival [13]. Interestingly, low-grade B-cell lymphoma has been demonstrated in up to 1.6% of retrieved femoral heads removed at the time of primary total hip replacement. However, the proportion of these patients developing clinically significant disease is felt to be extremely low (0.1%) [14].

Whilst the aetiology of PBL remains unknown, associations have been made with viral agents including human immunodeficiency virus and Epstein-Barr virus [15, 16]. Furthermore, associations have been reported between PBL and chronic inflammation, for example, relating to joint prostheses and rheumatoid arthritis [11, 12, 17].

Regarding the prognosis of PBL, an increase in reported overall five-year survival has been seen, from 46% in 1994 to 74% in 2013 [3, 5]. Factors associated with favourable prognosis include younger age, female sex, lack of B symptoms, and normal LDH levels [6]. As PBL is potentially curable, it must be differentiated from other primary bone malignancies and metastatic deposits by means of combined imaging modality and histological analysis.

Whilst the optimal treatment regimen for PBL remains unclear, a combination of R-CHOP-based chemotherapy plus radiotherapy has been associated with improved survival [2, 6, 8, 18]. The role of surgery in PBL remains controversial, but it appears that surgery is primarily utilised with the aim to restore function, particularly in the case of pathological fracture, and should not unduly delay medical treatment [3]. Surgical stabilisation as primary treatment has not been associated with improved survival [13]. Regarding radiotherapy in the case of pathological fracture, whilst retrospective analysis has found primary irradiation of the fractured bone to be associated with poorer response to subsequent chemotherapy [13], the authors of one prospective trail also reported that radiotherapy may prevent disability resulting from fractures associated with local recurrence [19]. Thus, in cases of pathological fracture, primary chemotherapy followed by consolidation radiotherapy to the fractured bone is generally regarded as mainstay of treatment.

The present study reports a case of an elderly gentleman found to have DLBCL affecting the proximal femur and associated acetabulum. He underwent surgical resection and reconstruction prior to commencing six cycles of R-Mini-CHOP chemotherapy.

## 2. Case Presentation

An 89-year-old gentleman with no significant past medical history presented to the emergency department (ED) of a district general hospital with atraumatic right hip pain and inability to weight-bear. The patient had a long history of right hip osteoarthritis causing pain and functional limitation, for which he had been referred by his general practitioner to the orthopaedic team for consideration of total hip replacement three years previous. However, the pain experienced during the week preceding ED presentation was much more severe in character. Of note, he had been seen by his general practitioner in the week preceding ED attendance with intense right hip pain on weight-bearing and passive movement and had been referred to the elderly care team with a view to optimising pain management.

**Figure 1:**
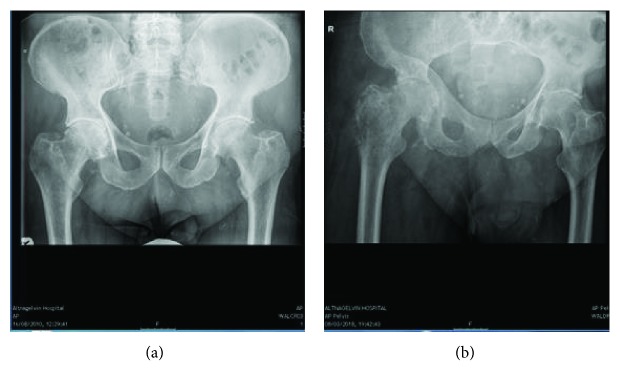
Plain film radiography compares pelvic radiographs from 2010 (a) and 2018 (b). On (a), the presence of moderately severe osteoarthritic change in the right hip is evident. On (b), there is a lytic lesion within the right femoral neck, intertrochanteric region, and proximal femoral metaphysis. Cortical breach with progressive sunburst periosteal reaction is evident.

Plain film radiography obtained in the emergency department (Figure 1) demonstrated a lytic lesion within the right femoral neck, intertrochanteric region, and proximal femoral metaphysis with evidence of cortical breach and progressive sunburst periosteal reaction.

**Figure 2:**
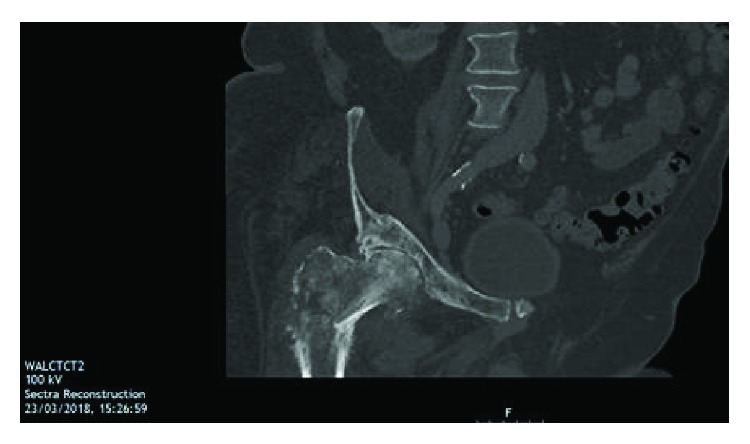
Computed tomography, coronal plane, demonstrating a bony lesion affecting the right acetabulum and proximal femur with associated proximal femoral fracture.

**Figure 3:**
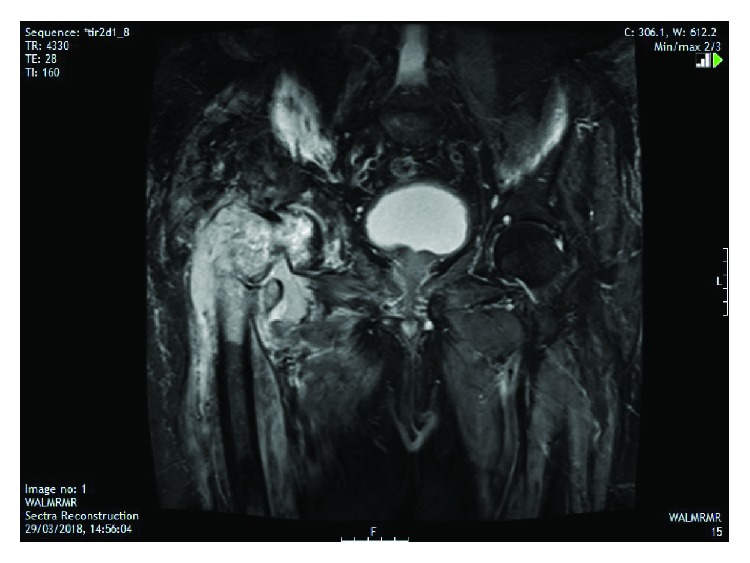
Magnetic resonance imaging demonstrating hyperintense marrow replacement, pathological fracture, and extraosseous soft tissue mass.

**Figure 4:**
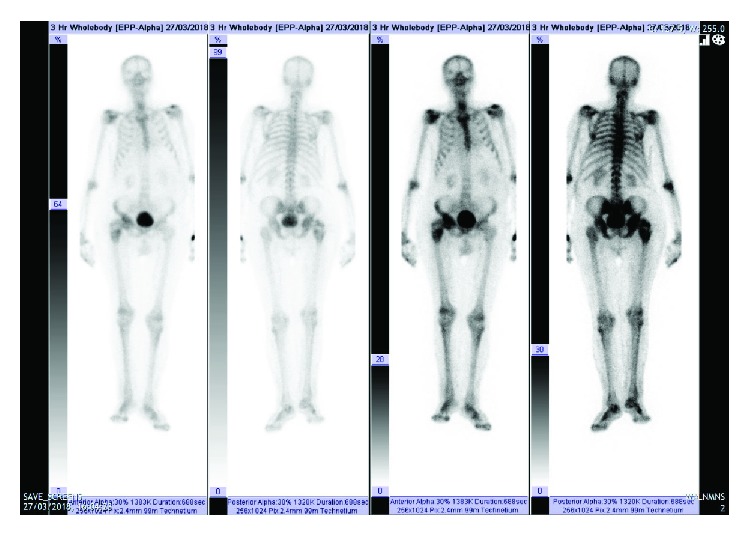
On the nuclear medicine bone scan, avid abnormal uptake is seen in the right acetabulum and proximal right femur, with a central more photopenic area in keeping with the tumor, pathological fracture, and soft tissue lesion.

The patient was admitted for further investigation and assessment. Computed tomography (CT) of the chest, abdomen, and pelvis (Figure 2) confirmed a bony lesion affecting both the right acetabulum and proximal femur with pathological intertrochanteric fracture and abnormal surrounding soft tissue. Magnetic resonance imaging (MRI) (Figure 3) findings were felt to be consistent with that of a primary bone tumor; tumoral necrosis was evident, and thigh muscles demonstrated oedema to the knee level. No lymphadenopathy was seen. Nuclear medicine scanning (Figure 4) demonstrated avid uptake in the right acetabulum and proximal femur with cortical breakthrough and marked extension to the surrounding thigh compartments but no uptake suggestive of distant disease. Fluorodeoxyglucose-positron emission tomography (FDG-PET) was not performed preoperatively.

Histological analysis of needle core biopsies obtained via a direct lateral approach under ultrasound guidance of the right femur was undertaken; the findings of which were in keeping with that of diffuse large B-cell lymphoma (DLBCL). Sections through the cores demonstrated tissue infiltration by large round blue cells staining positively for leukocyte common antigen, CD20, BCL2, and BCL6 and negatively for CD10, CD3, CD5, and cyclin-D1. The Ki67 proliferation index was high at approximately 80%. Interface fluorescent in situ hybridisation detected a BCL2 translocation but no BCL-6 or c-myc translocations.

This gentleman was transferred to Northern Ireland's Regional Trauma Centre where he underwent proximal femoral replacement with acetabular reconstruction, owing to the presence of pathological fracture as visualised on CT. Pathological analysis of the operative specimen demonstrated a tumor present within the medullary cavity with cortical, periosteal, and soft tissue involvements. The osseous resection margin was clear; however, the soft tissue resection margin was involved laterally. Histological analysis confirmed DLBCL of germinal centre phenotype staining positive for CD20, BCL6, and BCL2 and negative for CD3, CD5, CD10, MUM-1, cyclin-D1, C-MC, and TdT. The proliferation index was again high with MIB-1 of over 90%.

The patient received postoperative care in the intensive care unit where he required inotropic support ahead of ward transfer. FDG-PET undertaken two weeks postprocedure demonstrated high FDG uptake at the surgical site, but metabolically active disease at other sites was not seen.

He was discussed at the haematological multidisciplinary meeting postoperatively, and the diagnosis diffuse large B-cell lymphoma (DLBCL), NOS (M9680/3), was agreed. He was deemed stage IEA due to the involvement of contiguous bones. The patient embarked upon six cycles of R-Mini-CHOP (a regimen of rituximab with decreased dose cyclophosphamide, doxorubicin, vincristine, and prednisolone [18]), a regimen which is in widespread use within the unit amongst those greater than 80 years of age.

Throughout his R-Mini-CHOP treatment, the gentleman was able to fully weight-bear and made excellent progress with the rehabilitation team. Radiographically, implant alignment was satisfactory, and there was no evidence of prosthetic loosening. The patient subsequently declined radiotherapy treatment. Repeat FDG-PET was conducted five months following diagnosis; no further disease was demonstrated.

## 3. Discussion

To our knowledge, this is the first reported case of DLBCL affecting both the proximal femur and adjacent acetabulum. We suspect that the lesion initiated in the proximal femur and, through direct growth, encroached upon the acetabular margin. No separate disease foci were identifiable on imaging. We note that this gentleman has a long history of hip osteoarthritis. Associations between rheumatoid arthritis and DLBCL have been made [11]; however, to date, no studies have reported association between osteoarthritis and DLBCL. Given the well-documented “wear and repair” nature of osteoarthritis [20] involving proinflammatory cytokines, many of which have been implicated in oncogenic pathways, we feel that molecular studies to investigate such an association are warranted.

Owing to his good functional status, this patient was fit to undergo a somewhat major surgical procedure prior to proceeding with R-Mini-CHOP chemotherapy. To date, the role of surgery amongst patients with PBL remains controversial. No clear national guidelines exist regarding the management of PBL; however, we appear to have managed this gentleman in accordance with current literature. Despite chemoradiation being usually deemed the mainstay of treatment for low-stage disease, this gentleman underwent surgery to manage his pathological fracture and restore function without unduly delaying medical therapy [3].

We note that had the patient not preceded to operative fixation, he would have been required to remain nonweight-bearing or, at best, partial weight-bearing on the affected side throughout all six cycles of chemotherapy [21]. We propose that for a patient in their ninth decade of life, this restriction would have a severe negative impact on their overall health status and quality of life. Such physical restriction would result in the patient scoring higher on the Eastern Cooperative Oncology Group (ECOG) scale, and it has been shown that lower ECOG scores are associated with greater likelihood of complete response to chemotherapy and increased five-year overall survival rates [22]. Furthermore, the ECOG score forms part of the IPI clinical risk calculator score in combination with scores for age, Ann Arbor stage, serum lactate dehydrogenase, absolute lymphocyte counts, bulky disease, and sex, and low IPI scores has also been associated with increased five-year overall survival rates [22]. We suggest that should the patient not have been permitted to fully mobilise owing to fracture fixation, he would be at heightened risk of chest sepsis, particularly during the period of transient neutropenia associated with each R-Mini-CHOP cycle, and thrombotic events.

Additionally, prior to commencing R-Mini-CHOP, the patient received seven days of prephase steroids (prednisolone 100 mg orally). Prephase steroid use has been shown to improve baseline ECOG scores before chemotherapy and ameliorate the depth and length of neutropenia seen with the first cycle of R-CHOP [23]. Thus, with a combination of surgery and steroids, the patient who was initially nonmobile due to pain could be deemed to have an ECOG score of two.

Prephase steroids have also been associated with decreased therapy-associated mortality and decreased incidence of tumor lysis syndrome [23]. Concerns may be raised regarding the negative impact which high-dose prednisolone administration may have on tissue healing when commenced within the first few weeks postoperative. However, acute high-dose corticosteroids of less than 10-day duration have been found not to be associated with adverse effects on wound healing [24].

In short, to date, owing to small numbers of patients in limited case series, the impact of surgery pre- or postchemotherapy on survival cannot be determined. It is anticipated that with further research on the matter and collation of data from multiple centres, clearer guidance will emerge on this matter. The need for timely and regular communication between orthopaedic and haematology teams is paramount to ensure optimal patient care.

## 4. Conclusion

Doctors, particularly those working in the emergency department and in general practice, must maintain a high index of suspicion for malignancy when encountering elderly patients with atraumatic skeletal pain, including when the patient has no history of cancer and no systemic symptoms or other significant clinical findings. PBL should also not be forgotten when considering causes of isolated lyric lesions seen on imaging in such patients. It is crucial that such patients are investigated appropriately, and a diagnosis is made in a timely fashion to avoid delaying treatment.

Despite 90 years from first publication, ironically in the same year as our patient was born, no guidelines are in widespread clinical use regarding the optimum management of PBL, owing to the rarity of the condition, only small numbers of patients in each case series [6] and limited prospective study [19]. Multimodal therapy of R-CHOP chemotherapy plus radiotherapy, with surgical management in cases of fracture prophylaxis or fixation, appears to be associated with the best overall and progression-free survival [9]. It is anticipated that larger, robust clinical trials will shed further light on this matter. However, in the interim, each patient should be considered on an individual basis with interdisciplinary involvement to determine their suitability for each modality.
